# The PET-CT Deception: A Postoperative Retroperitoneal Mass Resembling Recurrent Burkitt Lymphoma Following Laparoscopic Colectomy

**DOI:** 10.7759/cureus.109580

**Published:** 2026-05-25

**Authors:** Zoi Lamprinou, Elisavet Kanna, Anna Ntanika, Ioannis Skondras

**Affiliations:** 1 2nd Pediatric Surgery Department, Athens General Children's Hospital Panagiotis and Aglaia Kyriakou, Athens, GRC

**Keywords:** abdominal lymphoma, oncology, pet-ct with 18f-fdg, postoperative lesion, primary burkitt lymphoma

## Abstract

Burkitt lymphoma (BL) is a highly aggressive B-cell non-Hodgkin lymphoma with excellent chemosensitivity when appropriately treated. However, the interpretation of post-operative imaging findings, particularly fluorodeoxyglucose positron emission tomography/computed tomography (18F-FDG PET/CT), remains challenging in distinguishing residual disease from benign post-therapeutic changes. We present the case of a 13-year-old adolescent with ileocecal BL who underwent successful chemotherapy followed by laparoscopic right hemicolectomy with intracorporeal anastomosis. Post-operative PET/CT imaging demonstrated a nodular lesion in the tumor resection bed with increased FDG uptake, raising concern for residual disease. However, adoption of active surveillance demonstrated progressive resolution of the lesion. This case highlights the importance of cautious interpretation of postoperative PET/CT findings, as hypermetabolic lesions may represent benign post-therapeutic changes rather than residual disease. In selected asymptomatic patients with favorable clinical evolution, close surveillance may be considered to avoid unnecessary invasive procedures or overtreatment.

## Introduction

Burkitt lymphoma (BL) is one of the most aggressive B-cell non-Hodgkin lymphomas, characterized by rapid proliferation and a high predilection for extranodal involvement [1]. Sporadic BL, the most common form in Western countries, accounts for approximately 1-2% of adult lymphomas and up to 30-50% of pediatric non-Hodgkin lymphomas, predominantly affecting children and adolescents, with a peak incidence between 10 and 20 years of age and a male predominance. The ileocecal region represents the most common site of gastrointestinal involvement [1]. Despite its aggressive biology, BL demonstrates remarkable chemosensitivity, and intensive short-course multiagent chemotherapy protocols such as the FAB (French-American-British) LMB (Lymphomes Malins B) and Berlin-Frankfurt-Münster (BFM)-based regimens, combined with surgical intervention when appropriate, achieve cure rates exceeding 80-90% in pediatric and adolescent populations [2].

However, the interpretation of residual masses detected after treatment remains a significant clinical challenge, as distinguishing viable residual disease from benign post-therapeutic changes such as inflammation, fibrosis, or necrosis is often difficult.

18F-FDG PET/CT is widely used for response assessment in lymphoma due to its high sensitivity. However, in the post-treatment setting, its specificity is limited, as increased FDG uptake may also reflect inflammatory or post-surgical changes rather than active malignancy. SUVmax (maximum standardized uptake value), a semiquantitative measure of FDG uptake intensity within a lesion, is commonly used to assess metabolic activity on PET imaging. While the negative predictive value (NPV) of PET/CT approaches 100% in most lymphoma subtypes, its positive predictive value is considerably lower due to frequent false-positive findings [3,4]. These limitations are particularly relevant following chemotherapy and surgery, where inflammatory responses and tumor necrosis can produce hypermetabolic lesions that closely mimic disease recurrence [5].

This diagnostic uncertainty has important clinical implications, as misinterpretation of imaging findings may lead to unnecessary invasive procedures or escalation to potentially toxic salvage therapy. In clinical practice, management decisions are therefore guided by a combination of imaging findings, clinical status, and interval changes on serial studies. In selected asymptomatic patients with favorable treatment response, a strategy of active surveillance may be considered prior to pursuing invasive diagnostic or therapeutic interventions.

We report a case of postoperative FDG-PET/CT uptake mimicking recurrent BL, which represented a benign inflammatory process, highlighting the risk of false-positive imaging in the early post-treatment setting and the value of cautious, context-driven management.

## Case presentation

A 13-year-old male presented with a one-month history of intermittent abdominal pain and diarrhea. One week prior to admission, he developed hematochezia associated with low-grade fever. Abdominal ultrasonography and cross-sectional imaging demonstrated a large, well-defined abdominal mass measuring approximately 9 cm, arising from the ileocecal region, with marked thickening of the terminal ileum, hypoechoic infiltrative areas, and loss of normal bowel wall stratification. Further diagnostic evaluation, including contrast-enhanced computed tomography, upper gastrointestinal endoscopy, and colonoscopy, established the diagnosis of high-grade BL involving the ileocecal valve. Baseline 18F-FDG PET/CT demonstrated markedly increased metabolic activity with an SUVmax of 23.

The patient exhibited the characteristic “starry sky” appearance on histology due to scattered histiocytes engulfing apoptotic lymphoma cells [6]. Molecular and immunophenotypic analysis revealed the pathognomonic t(8;14) translocation involving the MYC gene. The disease was staged as stage III - group B, high risk, according to the St. Jude staging system, given the presence of extensive intra-abdominal disease without bone marrow or central nervous system involvement.

The patient received intensive multiagent chemotherapy according to the FAB/LMB /Inter-B-NHL pediatric B-cell lymphoma protocols, consisting of vincristine, cyclophosphamide, prednisolone, rituximab, methotrexate, folinic acid, doxorubicin and hydrocortisone. Following chemotherapy, PET/CT demonstrated a marked metabolic response with reduction of SUVmax to 1.73, accompanied by a substantial decrease in tumor size on conventional imaging. In view of the favorable radiologic response, surgical resection was planned. A laparoscopic right hemicolectomy was performed with complete resection of the primary tumor and the involved bowel segment. The anastomosis was performed intracorporeally using a stapling device, and the operative course was uneventful. The patient made an excellent post-operative recovery and was discharged in stable condition.

**Figure 1:**
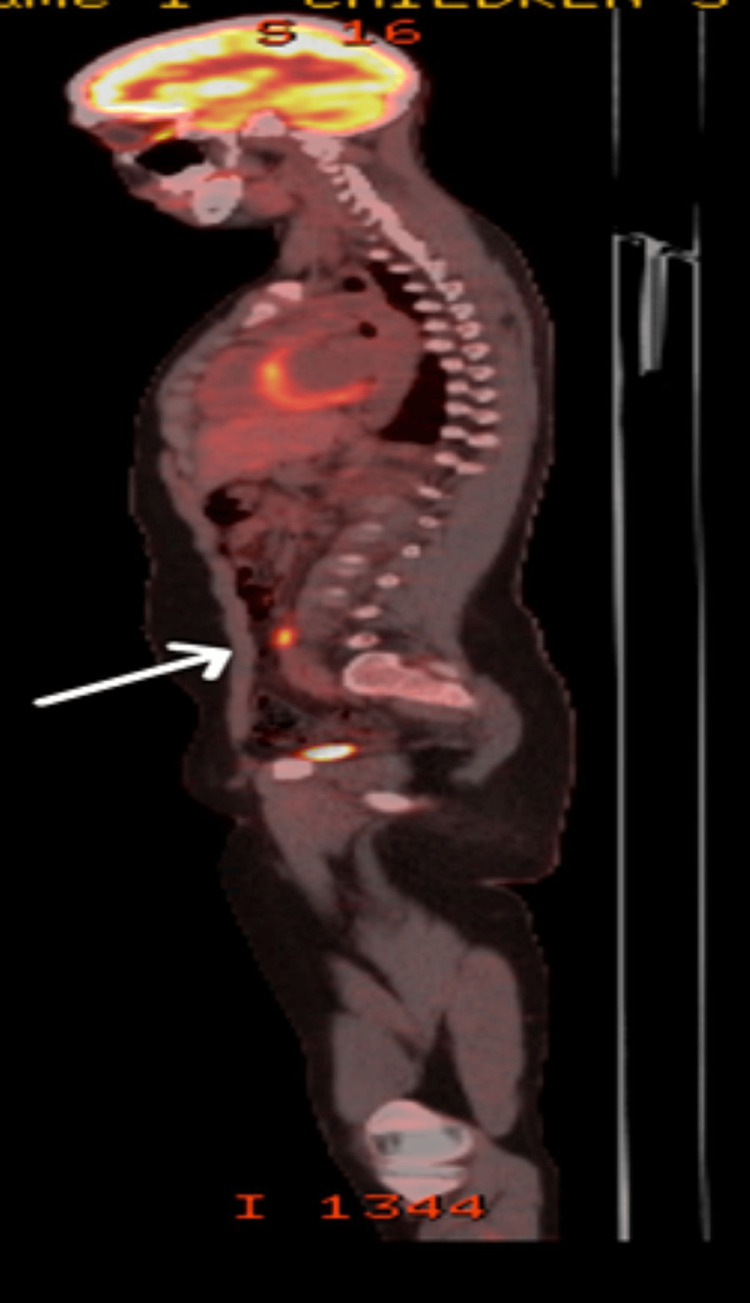
Axial fused 18F-FDG PET/CT image obtained four weeks postoperatively demonstrating focal hypermetabolic uptake within a nodular lesion adjacent to the right psoas muscle (arrow), initially concerning for residual or recurrent Burkitt lymphoma.

Four weeks after surgical resection, post-operative imaging surveillance was performed. A contrast-enhanced CT scan of the abdomen and pelvis was obtained to assess the post-operative anatomy and evaluate for residual disease. This imaging revealed a nodular lesion measuring 2 × 1.2 × 1.7 cm located in close proximity to the psoas muscle within the tumor resection bed. Subsequently, an 18F-FDG PET/CT scan was performed for metabolic characterization of this lesion. The PET/CT study demonstrated focal increased FDG uptake at the site of the nodular lesion, with an SUVmax of 4.4, raising initial concern for residual active disease (Figure 1).

At the time of imaging evaluation, the patient was entirely asymptomatic, afebrile, hemodynamically stable, tolerating oral intake normally, and without abdominal pain, weight loss, night sweats, or other constitutional symptoms. Physical examination revealed a soft, non-tender abdomen without palpable masses or signs of peritoneal irritation. Laboratory evaluation demonstrated persistently normal inflammatory markers, stable hemoglobin levels, normal leukocyte counts, and no biochemical evidence suggestive of active disease or infection. At this stage, the key clinical decision was whether to proceed with biopsy or initiate salvage therapy versus adopting a conservative surveillance approach. The incongruence between the patient’s excellent clinical status, the dramatic response to initial therapy, and the appearance of a new hypermetabolic lesion in the immediate post-operative period created a diagnostic dilemma. After multidisciplinary discussion involving pediatric hematology-oncology, surgical, and radiology teams, an active surveillance strategy was adopted rather than pursuing immediate histological confirmation through repeat biopsy or escalation to salvage chemotherapy. The clinical rationale for this conservative approach and to avoid biopsy was based on several factors: (1) the patient’s asymptomatic status with excellent clinical response to initial therapy; (2) the post-operative timing of the lesion appearance, suggesting a potential post-therapeutic reactive process; (3) the reported high incidence of false-positive PET/CT findings in the post-chemotherapy setting, particularly related to inflammatory and necrotizing processes [7].

**Figure 2:**
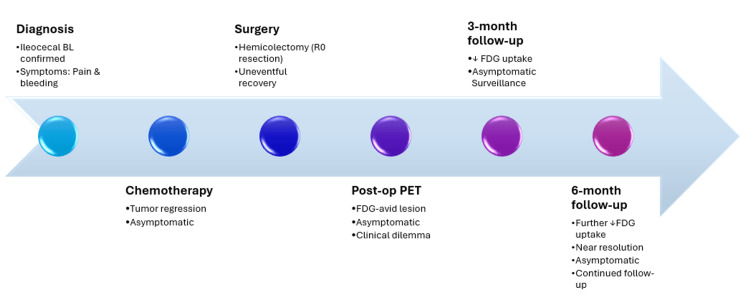
Timeline summarizing the patient’s clinical course, including initial presentation, diagnostic evaluation, chemotherapy, surgical resection, postoperative PET/CT findings, and serial imaging follow-up demonstrating progressive metabolic resolution of the hypermetabolic lesion. BL: Burkitt lymphoma

Follow-up imaging was performed using a structured surveillance protocol consisting of monthly abdominal ultrasonography and repeat PET/CT imaging at three and six months after surgery. The rationale for this approach was to allow close monitoring during the early post-operative period, when the risk of diagnostic uncertainty is highest, while spacing PET/CT studies to permit meaningful assessment of metabolic changes over time and to minimize radiation exposure. Over the subsequent follow-up period, the hypermetabolic activity associated with the nodular lesion demonstrated a progressive and sustained decrease in intensity. The clinical course involved several critical decision points where management was guided by the integration of imaging findings with the patient’s overall clinical status. The lesion itself remained stable in dimensions, and no new lesions appeared on subsequent imaging studies. Throughout follow-up, the patient remained clinically well, with stable physical examination findings, persistently normal laboratory parameters, and no development of constitutional symptoms or radiologic evidence of disease progression. This pattern of gradual metabolic resolution while maintaining radiographic stability was more suggestive of a benign post-therapeutic process, such as an inflammatory pseudotumor rather than true recurrent lymphoma. A structured timeline summarizing diagnosis, chemotherapy, surgery, postoperative PET/CT findings, and follow-up imaging is provided in Figure 2.

## Discussion

The management of post-treatment residual masses represents one of the most challenging aspects of lymphoma follow-up imaging. While advanced imaging technologies such as 18F-FDG PET/CT have improved our ability to identify metabolically active disease, the presence of FDG uptake alone is insufficient to confirm active malignancy, particularly in the immediate post-operative and post-chemotherapy periods [3]. The pathophysiology of false-positive PET/CT findings in this setting is multifactorial, including chemotherapy-induced tumor necrosis, inflammatory responses to tissue injury, recruitment of inflammatory cells, including macrophages and histiocytes, and post-operative granulation tissue formation [8].

An inflammatory pseudotumor, also known as an inflammatory myofibroblastic tumor or xanthomatous pseudotumor in certain contexts, represents a benign mesenchymal lesion composed of inflammatory cells and myofibroblastic spindle cells [9]. In the post-chemotherapy setting, these lesions can develop at sites of prior disease involvement and represent either reparative changes following tumor necrosis or florid inflammatory responses to chemotherapy-induced tissue injury [9]. Critically, these benign lesions can demonstrate significant FDG avidity, creating the appearance of active disease on PET/CT imaging [9]. The case reported by Mainolfi and colleagues in an adolescent male with prior BL is particularly instructive: a 14-year-old patient with a large ileal mass treated for BL developed persistent FDG uptake at the site of prior involvement despite dramatic reduction in tumor volume and asymptomatic clinical status [9]. Laparoscopic biopsy revealed xanthomatous pseudotumor with histiocytic elements (CD68+ CD163+), multinucleated giant cells incorporating cholesterin crystals, T-lymphocytes, and fibroblasts (SMA+) [9]. Importantly, the translocation t(8;14) was negative, confirming the absence of BL relapse. Serial PET/CT imaging demonstrated a progressive decrease in metabolic activity over subsequent months, supporting the benign nature of this post-therapeutic lesion.

Recent literature has provided important data on the diagnostic accuracy of 18F-FDG PET/CT in pediatric patients with abdominal BL. A seminal study by Riad and colleagues examining 28 pediatric patients with abdominal BL found that while the NPV of post-therapy PET/CT was 100%, the positive predictive value (PPV) was only 25% [3,4]. In all four patients with positive post-treatment PET/CT studies in this series, pathological examination revealed inflammatory changes without evidence of viable malignant cells, demonstrating the high false-positive rate [3]. Similarly, Carrillo-Cruz and colleagues, in their study of the role of 18F-FDG PET/CT in BL management, reported a post-therapy NPV of 100% but a PPV of only 20%, with false-positive lesions most commonly demonstrating reduced SUVmax (standardized uptake value) compared to initial diagnostic scans [4]. Using a cutoff value for SUVmax reduction of greater than 66% compared to initial diagnostic values significantly improved PPV to 100%, suggesting that quantitative analysis of FDG uptake reduction may aid in distinguishing true disease progression from post-therapeutic inflammatory changes.

The high false-positive rate of PET/CT in post-chemotherapy lymphoma patients has important therapeutic implications. Escalation to salvage chemotherapy or intensified therapy based solely on positive PET/CT findings in asymptomatic patients at the completion of initial therapy carries significant morbidity and toxicity risk [3]. Conversely, a negative PET/CT scan at the end of therapy provides strong reassurance regarding complete response, with reported NPV approaching 100% in multiple series [4,5].

The International Harmonization Project recommends that changing therapy on the basis of a positive FDG-PET/CT finding alone is not recommended in children with non-Hodgkin lymphoma, and biopsy confirmation is required in cases where therapeutic escalation is being considered [5]. Active surveillance with serial imaging represents a reasonable approach in asymptomatic patients with good performance status when clinical context and radiographic progression patterns are inconsistent with active malignancy [9]. In settings where PET/CT is not readily available, careful clinical evaluation combined with serial ultrasonography or contrast-enhanced CT may provide valuable information for monitoring such lesions and guiding management decisions.

In the context of BL specifically, additional factors support a conservative approach in carefully selected cases. Over 80% of patients with stage III BL and no disseminated disease at diagnosis respond to therapy with achievement of complete remission after the first three chemotherapy cycles [9]. When residual masses persist after initial therapy in asymptomatic patients, the probability that such residual disease represents viable malignancy ranges from only 10 to 20% [9]. Most commonly, histological examination reveals necrosis and/or fibrosis rather than active disease [9]. Furthermore, chemotherapy-induced inflammatory status may persist for several weeks or months after therapy completion, and as demonstrated in our case and the literature, such inflammatory lesions may demonstrate progressive resolution over time without additional intervention. Clinical factors supporting active surveillance in this case included: favorable clinical status, excellent initial treatment response, timing of imaging findings (lesion identified in the early post-operative period, when inflammatory changes are common), imaging characteristics (isolated lesion without evidence of widespread disease), lack of progression, and normal laboratory findings.

A key limitation of this case is the absence of histological confirmation of the lesion. Although the progressive resolution of metabolic activity on serial imaging strongly suggests a benign post-therapeutic process, the diagnosis of an inflammatory pseudotumor remains presumptive. Tissue biopsy would be required for definitive confirmation.

The differential diagnosis for a nodular mass in the post-operative tumor resection bed includes: (1) residual or recurrent BL; (2) chemotherapy-induced inflammatory pseudotumor; (3) post-operative granulation tissue or seroma; (4) abscess or post-operative infection; and (5) other benign post-operative changes, including fat necrosis or foreign body reactions [10]. The combination of clinical factors, complete asymptomatic status with normal laboratory values, dramatic initial chemotherapy response, and progressive resolution of metabolic activity on serial imaging, strongly favored a benign post-therapeutic process over active malignancy in this case.

This case underscores important considerations in interpreting post-treatment PET/CT findings in BL. FDG uptake does not necessarily indicate residual or recurrent disease, particularly in the early post-operative or post-chemotherapy period, when inflammatory changes are common. Clinical context is essential, as asymptomatic patients with a good therapeutic response and normal laboratory findings are less likely to have active disease. Given the risk of false-positive results, PET/CT findings should be interpreted cautiously and alongside clinical and radiological evolution. While biopsy remains the gold standard when suspicion persists, active surveillance with close follow-up may be appropriate in carefully selected patients, with decisions individualized based on clinical context and available resources.

## Conclusions

This case of ileocecal BL with a postoperative inflammatory pseudotumor highlights the challenges of interpreting post-treatment FDG-PET/CT findings. Hypermetabolic lesions identified after chemotherapy and surgery should be interpreted in the context of the patient’s clinical status, laboratory findings, treatment response, and longitudinal imaging evolution rather than in isolation.

In selected asymptomatic patients, postoperative FDG uptake may represent benign inflammatory change rather than recurrent disease, and careful surveillance with serial imaging may be considered before invasive procedures or treatment escalation. However, as histological confirmation was not obtained in this case, biopsy remains the standard approach when suspicion for recurrence persists or disease progression is observed.
